# Contraceptive Adoption, Discontinuation, and Switching among Postpartum Women in Nairobi's Urban Slums

**DOI:** 10.1111/j.1728-4465.2015.00038.x

**Published:** 2015-12-08

**Authors:** Joyce N. Mumah, Kazuyo Machiyama, Michael Mutua, Caroline W. Kabiru, John Cleland

**Affiliations:** ^1^ Associate Research ScientistAfrican Population and Health Research Center, Population Dynamics and Reproductive Health Program, APHRC Campus2nd Floor Manga Close, Off Kirawa RoadNairobiKenya; ^2^ Data AnalystAfrican Population and Health Research Center, Population Dynamics and Reproductive Health Program, APHRC Campus2nd Floor Manga Close, Off Kirawa RoadNairobiKenya; ^3^ Research ScientistAfrican Population and Health Research Center, Population Dynamics and Reproductive Health Program, APHRC Campus2nd Floor Manga Close, Off Kirawa RoadNairobiKenya; ^4^ Research Fellow, Faculty of Epidemiology and Population HealthLondon School of Hygiene & Tropical MedicineLondonUnited Kingdom; ^5^ Emeritus Professor of Medical Demography, Faculty of Epidemiology and Population HealthLondon School of Hygiene & Tropical MedicineLondonUnited Kingdom

## Abstract

Unmet need for contraception is highest within 12 months post‐delivery, according to research. Using longitudinal data from the Nairobi Urban Health and Demographic Surveillance System, we assess the dynamics of contraceptive use during the postpartum period among women in Nairobi's slums. Results show that by 6 months postpartum, 83 percent of women had resumed sexual activity and 51 percent had resumed menses, yet only 49 percent had adopted a modern contraceptive method. Furthermore, almost half of women discontinued a modern method within 12 months of initiating use, with many likely to switch to another short‐term method with high method‐related dissatisfaction. Women who adopted a method after resumption of menses had higher discontinuation rates, though the effect was much reduced after adjusting for other variables. To reduce unmet need, effective intervention programs are essential to lower high levels of discontinuation and encourage switching to more effective methods.

Estimates indicate that more than 215 million women who wish to postpone childbearing in low- and middle-income countries are not using any form of modern contraception (Singh et al. 2009). Discounting the partial protection from lactational amenorrhea, unmet need for family planning is particularly high among postpartum women (Ross and Winfrey 2001). Factors associated with nonuse of contraception include low educational and socioeconomic status, rural residence, and fertility goals (Westoff 2001). The consequences are high levels of unintended pregnancies, especially in low- and middle-income countries, with an estimated 73 million such pregnancies in 2012 (Sedgh and Hussain 2014). Unintended pregnancies have implications for the health and well-being of women and children, because a woman's risk of death increases with each successive pregnancy.

In Kenya, according to the 2008–09 Kenya Demographic and Health Survey (KDHS) (KNBS and ICF Macro 2010), 17 percent of births were unwanted and an additional 26 percent were mistimed. Low use of contraception and high levels of unmet need for family planning are largely responsible for the country's high incidence of unintended pregnancies. Unmet need for family planning in Kenya is higher within the extended postpartum period than at other times (Machiyama and Cleland 2014a), perhaps because women are still breastfeeding and the range of methods available after giving birth is limited. The 2008–09 KDHS shows that 23 percent of non-first births in Kenya occur less than 24 months apart, because by 12 months postpartum 92 percent of women had resumed sexual activity, 80 percent had resumed menses, yet only 44 percent were using any form of contraception (MCHIP and USAID 2012). It is well established that such short intervals pose a risk to the survival and health of infants (Tsui and Creanga 2009). Similarly, Ndugwa and colleagues (2011) investigated the degree to which women in Nairobi's urban slums protected themselves against pregnancy during the postpartum period and found that although women resumed sexual relations early—usually by the third month—many were not using any form of contraception up to 6 months postpartum. Furthermore, about 12 percent of these women were pregnant within 12 months of giving birth.

The range of contraceptive methods available after childbirth is a key determinant of adoption and use, as is the timing of return of menses (Salway and Nurani 1998a; Ali and Shah 2004; Gebreselassie, Rutstein, and Mishra 2008; Borda and Winfrey 2010; Rutstein and Winter 2014). A multicountry analysis by Ali and Shah (2004) showed that less than 30 percent of women adopt a method before the return of menses, while a study in Dhaka's slums showed that women were more likely to adopt a method after the resumption of menses (Salway and Nurani 1998a). In Peru and Indonesia, only half of modern methods were available to couples, and this factor was likely the reason for high use of periodic abstinence (Becker and Ahmed 2001). In Swaziland, half of the women who had reported use of condoms during the postpartum period had an unintended pregnancy (Warren, Abuya, and Askew 2013), indicating that use of short-term methods was often not effective in avoiding pregnancy. In Kenya, the vast majority of women practicing contraception used short-term hormonal methods (i.e., injectables and pills). Discontinuation of these methods is high, with a median duration of use of 9.8 and 15.4 months, respectively (Ali, Cleland, and Shah 2012). Moreover, only 30 percent of women who discontinued for method-related reasons switched to a modern method within three months of discontinuation (ibid.). Thus, both adoption of postpartum family planning and continuation of use have important policy and program implications.

Studies on postpartum contraceptive use have relied on cross-sectional data, with the notable exception of Salway and Nurani (1998a and 1998b) in Bangladesh. Moreover, most previous research on postpartum family planning focuses on the timing of contraceptive adoption, with limited examination of continuation of contraceptive use or switching after discontinuation. Our study draws on longitudinal data to address these gaps in evidence and to answer the following questions: (1) To what extent does postpartum contraceptive use overlap with lactational protection or postnatal abstinence? (2) Is the timing of contraceptive adoption (e.g., before or after resumption of menses) among women in urban slums related to length of use? (3) To what extent do baseline fertility preferences (e.g., want another child or not) predict successful prolonged use? and (4) To what extent do women promptly switch to another method after discontinuation? The longitudinal nature of our data is well suited for examining the dynamics of postpartum contraception and also implies greater accuracy in the dating of events than most other analyses that use retrospective data. Our data allow us to ascertain the temporal sequence of events—that is, resumption of sex and adoption of contraception in relation to resumption of menses.

## METHODS

### Study Setting

This study focuses on two Nairobi slums—Viwandani and Korogocho—where the African Population and Health Research Center (APHRC) runs the Nairobi Urban Health and Demographic Surveillance System (NUHDSS). As of December 2012, the NUHDSS—which collects and monitors health and demographic data among residents living in the two slums—covered approximately 66,000 people in about 27,000 households. Residents of these two slums face challenges that are often triggered by pervasive poverty and unmet service-provision needs, including poor housing, poor sanitation facilities, and poor health services. Of particular relevance to our study, the sexual and reproductive health outcomes on some indicators for women in slums are worse compared with their counterparts in broader Nairobi. For example, the maternal mortality ratio (MMR) in slums is estimated at 709 deaths per 100,000 live births (APHRC 2002), almost double the national ratio, estimated at 488 per 100,000. A study by Ziraba and colleagues (2009) found that most maternal deaths in slums occurred because labor and abortion were not managed properly, with less than 50 percent of women seeking care at a health facility.

### Data

Our study draws on data from the Maternal and Child Health component of a broader project entitled "Urbanization, Poverty, and Health Dynamics," which was nested in the NUHDSS. All women residing in NUHDSS sites who gave birth since September 2006 and their children were enrolled in the project. Follow-up visits were carried out every four months. Our study uses data on women who were interviewed at least three times and thus provide a minimum of 12 months of information. The first baseline observations (Cohort 1 and Wave 1) took place between February and April 2007, with follow-up visits and new recruitments made routinely thereafter. Overall, 5,345 women were recruited by the end of the study in October 2010. Data on reproductive events such as breastfeeding, postpartum amenorrhea and abstinence, contraceptive use, sexual behavior, and fertility intention were collected since the birth of the index child using a monthly calendar method. Interviews were conducted in Swahili, and trained fieldworkers recruited mothers and their children during each visit; these mothers formed new cohorts, while updated information was obtained from mothers recruited in the previous cohort. We use data from seven cohorts of women collected since February 2007. Table 1 shows relatively high attrition across the waves, an expected feature of surveillance in a slum population. For example, of the 363 women who were enrolled in the first cohort, only 148 were reinterviewed during the tenth visit. In this regard, out of the 10 cohorts of women interviewed during 2007–10, only 7 cohorts (1–6 and 8) had sufficient numbers of women with 12 months of continuous calendar data to be included in our analysis (N=3,579). As a result of attrition and our selection criterion, we assessed the sample to ascertain whether

**TABLE 1 Number of women interviewed during 2007–10, with at least 12 months of continuous calendar data since birth of index child**

| | Wave 1[a] | Wave 2[a] | Wave 3 | Wave 4 | Wave 5 | Wave 6 | Wave 7 | Wave 8 | Wave 9 | Wave 10 | Total |
|---|---|---|---|---|---|---|---|---|---|---|---|
| Cohort 1[a] | 363 | 374 | 370 | 337 | 296 | 262 | 220 | 198 | 177 | 148 | 2,745 |
| Cohort 2 | — | 281 | 280 | 274 | 254 | 237 | 224 | 211 | 184 | 167 | 2,112 |
| Cohort 3 | — | — | 481 | 475 | 463 | 438 | 414 | 391 | 360 | 398 | 3,420 |
| Cohort 4 | — | — | — | 696 | 689 | 670 | 608 | 549 | 423 | 379 | 4,014 |
| Cohort 5 | — | — | — | — | 331 | 332 | 331 | 303 | 261 | 208 | 1,766 |
| Cohort 6 | — | — | — | — | — | 606 | 609 | 601 | 534 | 414 | 2,764 |
| Cohort 8 | — | — | — | — | — | — | — | 478 | 479 | 446 | 1,403 |
| Total | 363 | 655 | 1,131 | 1,782 | 2,033 | 2,545 | 2,406 | 2,731 | 2,418 | 2,160 | 18,224 |

[a]Cohort 1 (Waves 1 and 2): 11 women interviewed in Wave 2 did not have calendar data in Wave 1.
NOTE: Cohorts 7, 9, and 10 had very few women recruited into the study and few women with 12 months of continuous data.

women retained for analysis were statistically different from those excluded. After checking for differences, we found that the characteristics of women retained in our analysis were not very different from those of the women not included (not shown). However, a slightly higher proportion of women reported being Kamba, had completed primary education, and were primiparous, compared with the women retained for analysis.

Ethical approval for the NUHDSS and Urbanization, Poverty, and Health Dynamics (UPHD) research programs was received from the Ethical Review Board of the Kenya Medical Research Institute.

### Analysis

Survival analysis was used to estimate the probability of adoption of first contraceptive method in the first 12 months postpartum. We further computed discontinuation rates based on all women who adopted a family planning method. While survival analysis on time to adoption uses woman-months since the birth of the index child observed, cumulative discontinuation rates are based on episodes of use of any contraceptive method. An episode in this study is defined as a period of uninterrupted use (in months) that might still be ongoing (Ali, Cleland, and Shah 2012). Discontinuation rates are presented at 3, 6, and 12 months postpartum. We compute hazards of adopting a modern contraceptive method and of discontinuation of any contraceptive method using the Cox proportional hazard model. In analyzing adoption of the first modern method, we use mother's age at birth of index child, marital status at recruitment, level of education at recruitment, parity, baseline fertility intention, site, and ethnicity as explanatory variables. In analyzing discontinuation, in addition to the above-mentioned explanatory variables, we also use timing of contraceptive adoption, type of contraception, and breastfeeding status as explanatory variables. To assess possible problems of collinearity, we ran separate discontinuation analyses with fertility intention and parity. Results showed no significant differences, so we present the results with both variables included. We used life table methods to estimate the reproductive status of women at 1, 3, and 6 months after discontinuation due to method-related or unspecified reasons, thereby excluding cases where discontinuation reflected the desire for another child. For the switching analysis, among the 844 women who discontinued the first postpartum contraceptive method for method-related or unstated reasons, data were missing for 128 women at 6 months after discontinuation and were therefore excluded from the analysis, leaving 716 women.

To assess the timing between contraceptive use and resumption of menses and of sexual activity since birth of the index child, we classify the monthly calendar into ordinal month since the birth of the index child, using the classifications of mutually exclusive categories of protection and risk with regard to contraceptive use as defined by Ndugwa and colleagues (2011). Protection and risk categories include the following. *Protected*: Months a woman is considered protected if she has not yet resumed sexual activity (irrespective of whether she is amenorrheic). *Partially protected*: A woman is considered to have partial protection during months she is amenorrheic but sexually active, since she is at risk of getting pregnant. *Exposed*: Months of exposure includes months where a woman is not amenorrheic and is sexually active. These months are considered protected if the woman was using any type of modern contraceptive method. *Currently pregnant*: Defined as months of pregnancy after birth of the index child.

### RESULTS

Table 2 shows the summary of selected background characteristics for the 3,579 women with 12 months of continuous data from the seven cohorts in our sample. About 53 percent of the

**TABLE 2 Background characteristics of women at recruitment during 2007–10, based on all women having at least 12 months of continuous calendar data since birth of index child**

| Characteristic | Percent | Number of women |
|---|---|---|
| Site | | |
| Korogocho | 52.9 | 1,892 |
| Viwandani | 47.1 | 1,687 |
| Mother's age at birth | | |
| 15–24 | 55.9 | 1,999 |
| 25–34 | 36.7 | 1,315 |
| 35–49 | 7.4 | 265 |
| Mother's marital status at recruitment | | |
| Currently married/cohabiting | 83.4 | 2,986 |
| Previously married | 6.1 | 220 |
| Never married | 10.4 | 373 |
| Mother's level of education at recruitment | | |
| No education/incomplete primary | 29.8 | 1,066 |
| Complete primary | 46.4 | 1,660 |
| Secondary + | 23.3 | 833 |
| Missing | 0.6 | 20 |
| Ethnicity | | |
| Kikuyu | 26.5 | 950 |
| Kamba | 19.6 | 700 |
| Luo | 18.9 | 676 |
| Luhya | 17.8 | 636 |
| Other | 17.2 | 617 |
| Parity | | |
| 1 | 28.9 | 1,034 |
| 2 | 25.2 | 903 |
| 3 | 15.8 | 564 |
| 4 and above | 20.5 | 732 |
| Missing | 9.7 | 346 |
| Baseline fertility intentions[a] | | |
| Wanted more | 56.7 | 2,021 |
| Wanted no more | 32.1 | 1,145 |
| Undecided/missing/not asked[a] | 11.2 | 399 |
| Total | 100.0 | 3,579 |

[a]Baseline prospective fertility intention was not asked of pregnant women.

women resided in Korogocho. The majority of women were between ages 15 and 24 (56 percent), and most women reported being in a marital union or living with a partner at the time of recruitment (83 percent). Nearly half had completed primary school (46 percent). The most common ethnic group was Kikuyu (27 percent). About 29 percent of women were primiparous, 25 percent had two children, and 21 percent had 4 or more children. At the first interview, more than half (57 percent) wanted more children.

### Time to Menstrual, Sexual, and Contraceptive Use Resumption

Using survival analyses, we employ monthly calendar data to estimate the first occurrence of modern contraceptive use, initiation of sexual activity, and resumption of menses since the birth of the index child. Figure 1 shows the survival curves for time to first menses, first use of modern contraception, and sexual resumption by ordinal postpartum month. By 6 months postpartum, about 49 percent of women in our sample had resumed menstruation. About 56 percent to 83 percent of women had resumed sexual relations by 3 and 6 months postpartum, respectively, suggesting that sexual activity typically preceded resumption of menses. Finally, 49 percent of women in the sample initiated modern contraceptive use at 6 months postpartum. Survival probabilities for all three events show that by 12 months postpartum, more than 90 percent of the women had resumed sexual relations, only 30 percent had not yet resumed menses, and about 60 percent had initiated use of modern contraception.

### Menstrual Resumption, Sexual Activity, and Contraceptive Use by Months Since Birth

Table 3 provides summary data for various categories of monthly postpartum protection and pregnancy risk, defined by contraceptive use, sexual activity, and menstrual and pregnancy sta-

**FIGURE 1 Number of months to menstrual resumption, first use of modern contraception, and resumption of sexual relations among postpartum women in Nairobi's slums, 2007–10**

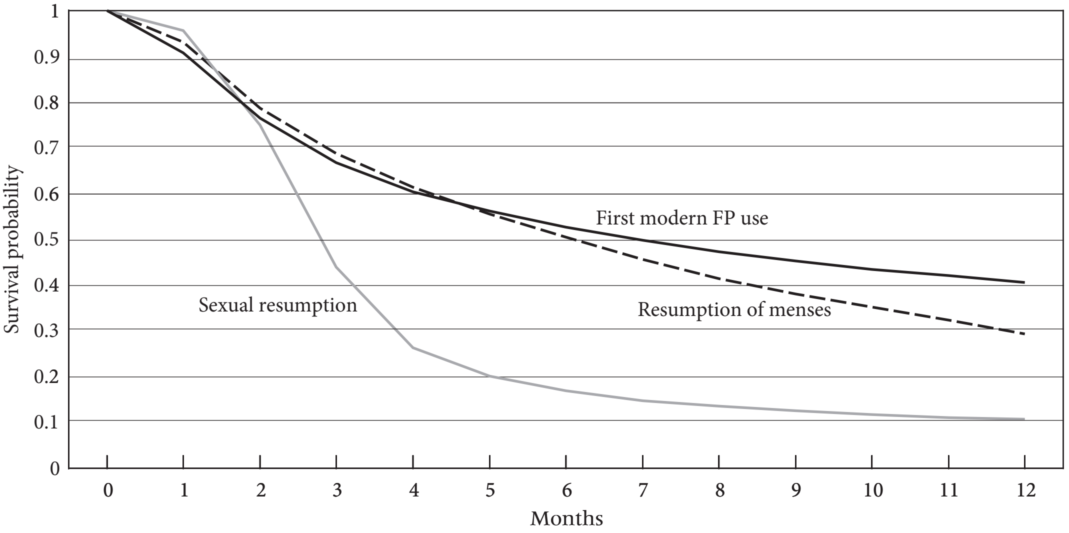

**TABLE 3 Percent distribution of woman-months of exposure to and protection against pregnancy among postpartum women in Nairobi's slums, by months since birth of index child, 2007–10**

| | 1–3 months | 4–6 months | 7–9 months | 10–12 months | Total | Total woman-months of observation |
|---|---|---|---|---|---|---|
| 1. FP + No menses + No sex [protected] | 4.9 | 2.3 | 0.9 | 0.6 | 2.2 | 932 |
| 2. FP + No menses + Sex [protected] | 2.3 | 13.1 | 12.6 | 10.6 | 9.7 | 4,148 |
| 3. FP + Menses + No sex [protected] | 2.2 | 2.8 | 2.2 | 2.5 | 2.4 | 1,033 |
| 4. FP + Menses + Sex [protected] | 1.9 | 19.2 | 29.0 | 34.9 | 21.3 | 9,125 |
| 5. No FP + No menses + No sex [protected] | 78.7 | 21.4 | 10.5 | 7.0 | 29.4 | 12,619 |
| 6. No FP + No menses + Sex [partial protection] | 4.8 | 25.7 | 22.7 | 17.8 | 17.8 | 7,627 |
| 7. No FP + Menses + No sex [protected] | 4.5 | 6.5 | 7.5 | 8.9 | 6.8 | 2,938 |
| 8. No FP + Menses + Sex [exposed] | 0.8 | 8.9 | 14.0 | 15.9 | 9.9 | 4,252 |
| 9. Currently pregnant | 0.0 | 0.1 | 0.6 | 1.9 | 0.6 | 274 |
| Total | 100.0 | 100.0 | 100.0 | 100.0 | 100.0 | 42,948 |
| Number of woman-months | 10,737 | 10,737 | 10,737 | 10,737 | 42,948 | |

tus, for the first 12 months since the birth of the index child. A total of 42,948 woman-months were observed. Only 0.6 percent of the woman-months comprised pregnancy, though this is likely be an underestimate because of underreporting of first-trimester pregnancies. Over the whole 12-month postpartum period, 36 percent of months were protected by contraception and 36 percent by sexual abstinence. Partial protection from amenorrhea accounted for about 18 percent of months, and 10 percent were unprotected. As expected, the roles of contraception, abstinence, and amenorrhea change radically during the 12-month period. In the first three months postpartum, sexual abstinence (without contraception) dominates, accounting for more than 80 percent of woman-months. By months 10–12, this contribution has shrunk to about 16 percent. The contribution of partial protection from lack of menses after resumption of sex peaks at 26 percent of woman-months at 4–6 months postpartum and falls to about 18 percent by months 10–12. The proportion of woman-months protected by use of contraception increases steadily from 11 percent in the first three months to 49 percent in the final three months. Despite this rise in contraceptive protection, full exposure to risk of pregnancy grows from less than 1 percent in the first three months to about 9 percent, 14 percent, and 16 percent over succeeding three-month segments.

Table 3 also shows overlaps between different protective states. As noted earlier, contraceptive use accounts for about 36 percent of all months in the first postpartum year. Of this total, 5 percent was redundant because of protection from abstinence and a further 10 percent partially redundant because of amenorrhea. Thus, out of the total of 15,238 woman-months of contraception (sum of last column in rows 1–4), 6,113 months, or 40 percent, were totally or partially redundant. This total or partial redundancy is removed when contraceptive use does not overlap with protection from abstinence or amenorrhea.

### Timing of First Contraceptive Adoption and Menstrual Resumption during the Postpartum Period

The timing of contraceptive adoption in relation to resumption of sexual intercourse and menses has implications for avoiding pregnancy during the postpartum period. Figure 2 shows that initiation of contraception during the postpartum period peaked during the month of the first menstrual cycle. About 22 percent of all adopters of a modern method started use in this

**FIGURE 2 Resumption of menses and timing of first modern contraception and first sexual intercourse**

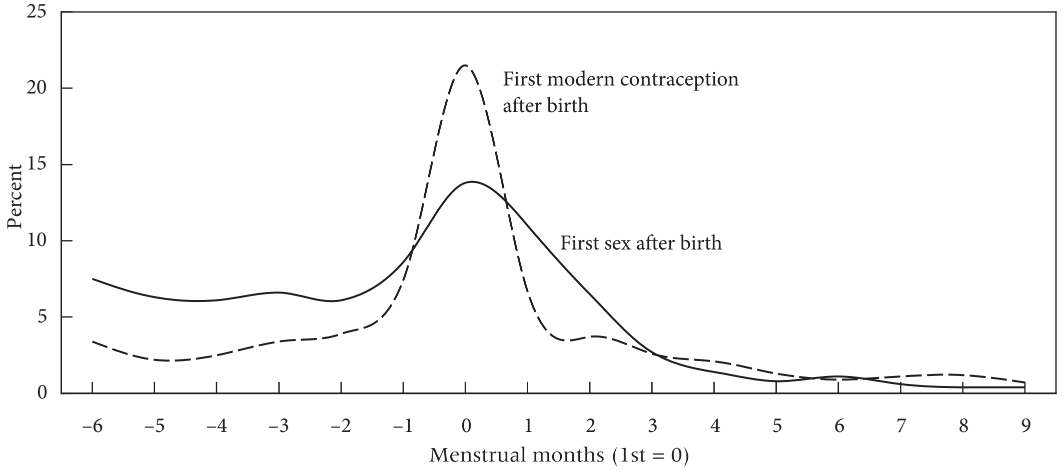

month, compared with less than 5 percent in the months preceding or following first menses. Although initiation of sexual activity also tended to peak around menstrual resumption, the peak was lower compared with contraceptive use, and initiation of sexual activity was more likely than contraceptive use to precede resumption of menses.

Results (not shown) indicate that, overall, injectables and pills were the most common choice of contraception during the postpartum period, especially after resumption of menses. Use of traditional methods was about the same during amenorrhea and after resumption of menses, with slight uptake observed after resumption of menses. Condom use was quite low during the 12 months postpartum, with only 6 percent reporting use during the post-amenorrhea periods. Long-acting methods such as implants, IUDs, and female sterilization were the least adopted methods during the 12-month postpartum period.

Analysis showed that the correlates of postpartum uptake of all methods, including traditional ones, were very similar to the correlates of uptake of a modern method. Thus the results presented here relate to modern methods. Table 4 provides the unadjusted and adjusted odds of modern contraceptive adoption at any time in the first year following childbirth. Results show that compared with women in Korogocho, women in Viwandani were about 1.3 times more likely to have adopted a modern contraceptive method during the 12-month postpartum period. Significant differences were observed by marital status: formerly married women and never-married women were 49 percent and 68 percent less likely, respectively, to adopt a modern contraceptive method during the 12-month postpartum period. Similarly, women aged 35–49 years had significantly lower odds of adopting a contraceptive method than women aged 15–24 years. Women with secondary or higher education and women who completed primary education were 1.3 times and 1.2 times more likely, respectively, to adopt a modern contraceptive method compared with women having incomplete primary or no formal education. With respect to parity, only women with four or more children reached the threshold for statistical significance and were 32 percent less likely to have adopted a method compared with women who had only one child. Significant differences were also observed by ethnic group: compared

**TABLE 4 Odds of adopting a modern method of contraception 12 months after birth among all women recruited during 2007–10, based on women with at least 12 months of continuous calendar data**

| | Hazard ratio | |
|---|---|---|
| | **Bivariate model** | **Multivariate Cox PH model** |
| Site (r = Korogocho) | | |
| Viwandani | 1.519*** | 1.266*** |
| Mother's age at birth (r = 15–24) | | |
| 25–34 | 0.982 | 0.980 |
| 35–49 | 0.529*** | 0.585*** |
| Mother's marital status at recruitment (r = Currently married/cohabiting) | | |
| Previously married | 0.526*** | 0.509*** |
| Never married | 0.366*** | 0.323*** |
| Mother's level of education at recruitment (r = No education/incomplete primary) | | |
| Complete primary | 1.499*** | 1.157** |
| Secondary+ | 1.678*** | 1.245*** |
| Ethnicity (r = Kikuyu) | | |
| Luhya | 0.824** | 0.766*** |
| Luo | 0.591*** | 0.596*** |
| Kamba | 0.944 | 0.730*** |
| Other | 0.606*** | 0.571*** |
| Baseline fertility intention (r = Wanted more) | | |
| Wanted no more | 0.940 | 1.137* |
| Undecided/missing/not asked | 0.945 | 1.020 |
| Parity (r = 1) | | |
| 2 | 1.103 | 0.977 |
| 3 | 1.079 | 0.916 |
| 4 and above | 0.674*** | 0.680*** |
| Missing | 0.815* | 0.991 |

*Significant at $p < 0.05$; ** $p < 0.01$; *** $p < 0.001$. (r) = Reference category. PH = Proportional hazards.

to Kikuyu women, women from all other ethnic groups had significantly lower odds of adopting a contraceptive method during the 12-month postpartum period. Women who reported having no desire for more children at baseline were 1.1 times more likely to adopt a modern contraceptive method compared with women who reported a desire for more children.

### Discontinuation of First Postpartum Contraceptive Method

Self-reported reasons for stopping use of the first method showed that method-related factors, such as side effects and health concerns, were dominant (50 percent; results not shown). Just over 10 percent stopped because of infrequent sex, 4 percent reported method failure, and 3 percent had no further need for protection because of the desire for another child or other reasons. We further assess the dynamics of contraceptive use following the initiation of any contraceptive method to estimate the length of use of methods. Table 5 shows the cumulative discontinuation rate for all reasons, per 100 episodes of first contraceptive use, by selected respondent characteristics among women who had adopted any method. Overall, 19 percent of women had discontinued their first contraceptive method within 3 months of starting, 32 percent within 6 months, and 49 percent within 12 months. Women who adopted a method after resumption of menses had higher discontinuation rates, especially within 12 months (54 percent), compared with women who adopted a method before (47 percent) or at the same

**TABLE 5 Discontinuation of any first contraceptive method used postpartum, based on women with at least 12 months of continuous calendar data**

| | Cumulative discontinuation rate per 100 episodes (percent) | | | Number of FP adopters |
|---|---|---|---|---|
| | 3 months | 6 months | 12 months | |
| Timing of contraceptive adoption | | | | |
| Before resumption of menses | 19.2 | 32.0 | 46.5 | 1,178 |
| Same time as resumption of menses | 17.3 | 28.9 | 48.3 | 736 |
| After resumption of menses | 19.6 | 34.9 | 53.8 | 713 |
| Type of contraception | | | | |
| Pills | 30.6 | 48.7 | 64.2 | 561 |
| Injectables | 13.6 | 24.0 | 39.7 | 1,480 |
| Traditional methods | 16.6 | 34.5 | 60.1 | 346 |
| Implants | 2.0 | 5.3 | 13.2 | 98 |
| Condoms | 50.9 | 65.4 | 84.2 | 118 |
| Other | 8.3 | 26.7 | 50.4 | 24 |
| Breastfeeding status at first adoption[a] | | | | |
| Not breastfeeding | 22.3 | 37.7 | 62.0 | 142 |
| Breastfeeding | 18.5 | 31.6 | 48.3 | 2,477 |
| Site | | | | |
| Korogocho | 17.9 | 30.9 | 51.1 | 1,230 |
| Viwandani | 19.6 | 32.5 | 46.7 | 1,397 |
| Mother's age at birth | | | | |
| 15–24 | 20.9 | 34.2 | 51.2 | 1,494 |
| 25–34 | 16.5 | 28.9 | 46.7 | 984 |
| 35–49 | 12.9 | 28.3 | 38.9 | 149 |
| Mother's marital status at recruitment | | | | |
| Currently married/cohabiting | 17.5 | 30.5 | 46.6 | 2,332 |
| Previously married | 33.2 | 47.9 | 69.2 | 122 |
| Never married | 26.6 | 38.9 | 67.7 | 173 |
| Mother's level of education at recruitment[b] | | | | |
| No education/incomplete primary | 20.6 | 33.8 | 54.0 | 660 |
| Complete primary | 17.7 | 30.9 | 47.9 | 1,279 |
| Secondary + | 19.1 | 32.1 | 45.5 | 681 |
| Ethnicity | | | | |
| Kikuyu | 18.5 | 30.9 | 46.0 | 734 |
| Luhya | 16.5 | 30.2 | 50.4 | 478 |
| Luo | 16.9 | 30.9 | 54.1 | 433 |
| Kamba | 22.0 | 34.4 | 48.1 | 582 |
| Other | 19.2 | 32.5 | 47.7 | 400 |
| Baseline fertility intention[c] | | | | |
| Wanted more | 18.8 | 32.8 | 50.2 | 1,502 |
| Wanted no more | 18.2 | 29.4 | 44.1 | 836 |
| Undecided/missing/not asked | 21.2 | 34.9 | 53.6 | 284 |
| Parity | | | | |
| 1 | 22.3 | 34.6 | 51.3 | 752 |
| 2 | 18.9 | 34.2 | 48.7 | 708 |
| 3 | 14.3 | 27.3 | 46.2 | 445 |
| 4 and above | 15.6 | 28.2 | 44.7 | 484 |
| Missing | 22.5 | 32.5 | 54.3 | 238 |
| Total | 18.8 | 31.9 | 48.8 | 2,627 |

NOTE: Male/female sterilization (n=27), IUD users (n=11), and emergency contraceptive users (n=5) were excluded from the sample.
[a]Eight episodes do not have data on breastfeeding status at discontinuation.
[b]Seven episodes do not have data on education.
[c]Five episodes do not have data on baseline fertility intention.

time as resumption of menses (48 percent). Users of condoms and pills had the highest discontinuation rates, while implants were the least discontinued. For example, 51 percent of women who adopted condoms had discontinued use by 3 months, with the level reaching 84 percent by 12 months. Similarly, about 31 percent of women who had adopted pills had discontinued by 3 months, with the level reaching 64 percent by 12 months. Compared with the pill, discontinuation of the most commonly used method, injectables, is low, 40 percent by month 12.

Women who were not breastfeeding at the time of adoption had higher discontinuation rates than women who were breastfeeding. Compared with their married counterparts, formerly married and never-married women had substantially higher discontinuation rates. By 3 months, 33 percent of formerly married women and 27 percent of never-married women had discontinued a method, compared with 18 percent of married women. Similarly, by 12 months, 68 percent of never-married and 69 percent of formerly married women had discontinued a method, compared with 47 percent of their married counterparts. With regard to educational groups, discontinuation rates were similar at 3 and 6 months. However, at 12 months a higher discontinuation level was observed among women with incomplete primary or no formal education (54 percent), especially compared with women with secondary and higher education (46 percent). No substantial differences were observed by ethnicity, parity, and site. With regard to mother's age at birth, women aged 15–24 had substantially higher discontinuation probabilities at 12 months (51 percent) than women aged 35–49 years (39 percent).

Table 6 shows the hazard ratios of discontinuation of the first postpartum method. After controlling for other variables, women who adopted a method after resumption of menses were 13 percent more likely to discontinue, compared with women who adopted before resumption of menses. Results for marital status remained significant. After controlling for other variables, formerly married and never-married women were 2.4 times and 1.7 times more likely to discontinue a contraceptive method, compared with married women. Condoms had the highest odds of discontinuation, with women 1.7 times more likely to discontinue the method after adoption compared with pills. All other methods (injectables and traditional) were less likely to be discontinued, compared with pills. Women with secondary education and higher had slightly lower odds of discontinuing a method compared with their counterparts having no formal or incomplete primary education. Women who gave birth between ages 25 and 34 and ages 35 and 49 were also less likely to discontinue compared with women who gave birth between ages 15 and 24.

### Switching after Discontinuation for Method-Related or Unspecified Reasons

We analyzed method switching among women who reported discontinuing for reasons that implied dissatisfaction with their method, or for an unspecified reason, and who therefore still had a need for contraception. More than half (54 percent) switched within one month of stopping, 63 percent within three months, and 70 percent within six months (results not shown). Table 7 presents detailed results of method switching at three months after discontinuation for women who had discontinued for method-related or unspecified reasons, by contraceptive method used before and after discontinuation. Table 8 shows switching by selected respondent characteristics. Results show that overall, three months after discontinuation, 32 percent of women were at risk (had not switched to any other method) and 5 percent had become pregnant. Reproductive consequence for method-related discontinuation was highest for women who discontinued traditional methods, with 12 percent reporting a pregnancy (Table

**TABLE 6 Hazard ratio of discontinuation of first postpartum method, based on women with at least 12 months of continuous calendar data**

| | Hazard ratio |
|---|---|
| Timing of contraceptive adoption | |
| Before resumption of menses | 1.000 |
| Same time as resumption of menses | 1.024 |
| After resumption of menses | 1.128 |
| Type of contraception | |
| Pills | 1.000 |
| Injectables | 0.417*** |
| Traditional methods | 0.757** |
| Implants | 0.100*** |
| Condoms | 1.692*** |
| Other | 0.564 |
| Breastfeeding status at first adoption[a] | |
| Not breastfeeding | 1.000 |
| Breastfeeding | 0.966 |
| Site | |
| Korogocho | 1.000 |
| Viwandani | 0.965 |
| Mother's age at birth | |
| 15–24 | 1.000 |
| 25–34 | 0.848* |
| 35–49 | 0.677** |
| Mother's marital status at recruitment | |
| Currently married/cohabiting | 1.000 |
| Previously married | 2.407*** |
| Never married | 1.710*** |
| Mother's level of education at recruitment[b] | |
| No education/incomplete primary | 1.000 |
| Complete primary | 0.924 |
| Secondary + | 0.840* |
| Ethnicity | |
| Kikuyu | 1.000 |
| Luhya | 1.189 |
| Luo | 1.121 |
| Kamba | 1.076 |
| Other | 1.070 |
| Baseline fertility intention | |
| Wanted more | 1.000 |
| Wanted no more | 0.917 |
| Undecided/missing/not asked | 1.092 |
| Parity | |
| 1 | 1.000 |
| 2 | 0.970 |
| 3 | 0.944 |
| 4 and above | 1.008 |
| Missing | 1.036 |

*Significant at $p < 0.05$; **$p < 0.01$; ***$p < 0.001$.
NOTE: Male/female sterilization (n=27), IUD users (n=11), and emergency contraceptive users (n=5) were excluded from the sample.
[a]Eight episodes do not have data on breastfeeding status at discontinuation.
[b]Seven episodes do not have data on education.

7). Failure to switch to another method (combining women at risk and pregnant) was highest for injectable users (51 percent), followed by traditional method users (32 percent). The main choice for women who discontinued pills, condoms, and traditional methods was injectables. Injectable discontinuers mainly switched to pills (Table 7). Table 8 shows that switching was

**TABLE 7 Status at three months after discontinuing any method for method-related or unstated reasons, by contraceptive method used before discontinuation**

| Contraceptive method used before discontinuation | Status at 3 months (%) | | | | | | | | Total | (N) |
|---|---|---|---|---|---|---|---|---|---|---|
| | | | Switched to: | | | | | | | |
| | At risk | Preg-nant | Pills | Inject-ables | Con-doms | Implants | Traditional methods | Other methods | | |
| Pills | 20.7 | 2.5 | 2.9 | 60.6 | 0.4 | 3.3 | 6.2 | 3.3 | 100 | (241) |
| Injectables | 47.1 | 3.9 | 29.1 | 2.6 | 2.6 | 5.2 | 4.6 | 4.9 | 100 | (306) |
| Condoms | 16.3 | 7.0 | 9.3 | 37.2 | 11.6 | 4.7 | 9.3 | 4.7 | 100 | (43) |
| Traditional methods | 20.5 | 11.6 | 8.9 | 43.8 | 3.6 | 1.8 | 6.3 | 3.6 | 100 | (112) |
| Other | 42.8 | 0.0 | 21.4 | 7.1 | 7.1 | 14.3 | 0.0 | 7.1 | 100 | (14) |
| Total | 32.1 | 4.7 | 15.8 | 30.7 | 2.6 | 4.2 | 5.6 | 4.2 | 100 | (716) |
| (N) | (230) | (34) | (113) | (220) | (19) | (30) | (40) | (30) | (716) | |

**TABLE 8 Status at three months after discontinuing any method for method-related or unstated reasons, by women's characteristics**

| Characteristic | Status at 3 months (%) | | | Total | (N) | p-value for chi-square test |
|---|---|---|---|---|---|---|
| | At risk | Pregnant | Switched | | | |
| Timing of contraceptive adoption | | | | | | |
| Before resumption of menses | 31.5 | 2.5 | 65.9 | 100 | (355) | |
| Same time as resumption of menses | 29.7 | 7.0 | 63.2 | 100 | (185) | 0.076 |
| After resumption of menses | 35.1 | 7.0 | 57.9 | 100 | (171) | |
| Missing | | | | | (5) | |
| Baseline fertility intention | | | | | | |
| Wanted more | 31.1 | 5.7 | 63.2 | 100 | (424) | |
| Wanted no more | 34.6 | 2.0 | 63.4 | 100 | (205) | 0.027 |
| Undecided/not asked | 31.8 | 5.9 | 62.4 | 100 | (85) | |
| Missing | | | | | (2) | |
| Mother's level of education at recruitment | | | | | | |
| No education/incomplete primary | 40.3 | 5.8 | 53.9 | 100 | (191) | |
| Complete primary | 33.1 | 4.4 | 62.5 | 100 | (344) | 0.001 |
| Secondary+ | 20.7 | 4.5 | 74.9 | 100 | (179) | |
| Missing | | | | | (2) | |
| Total | 32.1 | 4.7 | 63.1 | 100 | (716) | |

lower among women who adopted a contraceptive method after resumption of menses and highest among women with secondary or higher education. The probability of switching was unrelated to fertility intentions.

### DISCUSSION

Many of the results from our study in two Nairobi slums are encouraging. Calibrated in terms of woman-months, protection from pregnancy by use of any method of contraception rose from the low level of 11 percent in the first three months to 37 percent in the following three months, and further to 45 percent and 49 percent. Postpartum contraceptive use in the Nairobi slums is considerably higher than national levels, estimated at 38 percent in the final three months of the first postpartum year (Winfrey and Rakesh 2014). The results from our study are lower than the estimate of 34 percent at three months postpartum for Kenya's urban population (ibid.).

Few women adopted a method before resumption of sex, and, in common with earlier analyses, we found that many awaited the return of menses before initiating use. At 4–6 months postpartum, the largest exposure category (26 percent of woman-months) was sexually active, amenorrheic women who were using no method. In the following three-month segments, the size of this category drops only modestly to 23 percent and then to 18 percent at months 10–12. Prevailing postpartum protocols recommend contraceptive protection for such women, except for those observing exclusive breastfeeding in the first six months postpartum. The risks of pregnancy during lactational amenorrhea are low, however, even in the second half of the postpartum year (Kennedy and Visness 1992).

Estimating the level of unmet need among postpartum women in Nairobi slums on the reasonable assumption that few women wish to become pregnant again within 12 months of a birth depends on the acceptability of the small, but not negligible, risks of conception before the return of menses. If these risks are deemed unacceptable, then unmet need is high. Over one-third (36 percent) of all woman-months were contributed by sexually active amenorrheic women who were using no method of contraception. However, only 10 percent of months were contributed by sexually active, menstruating women who were nonusers.

The complexity of postpartum contraception is increased by consideration of double or redundant protection and early discontinuation of use. In this slum population, menses certainly acted as a signal to start using a method, but contraceptive initiation before the return of menses was nevertheless common. We estimate that 40 percent of all contracepting months were totally or partially redundant because of sexual abstinence or amenorrhea. This high degree of overlap severely dilutes the impact of contraceptive practice on birth interval lengths.

If contraceptive use, once started, was prolonged, particularly among women initiating use before menses, overlap would be of little concern. However, contraceptive discontinuation rates were high, with almost half of women who adopted a method during the postpartum period discontinuing within 12 months, and with only a small difference between those who adopted before and after resumption of menses. The estimate from our study is much higher than the national estimate of 32 percent documented by the 2008–09 KDHS. The reason for this difference is unknown but in part may reflect differences in data collection. A prospective approach should be better suited than the retrospective approach used by the DHS to identify short episodes of use.

Unless followed by prompt method switching, this very high level of contraceptive discontinuation indicates that past users of contraception account for a large fraction of unmet need for contraception among women in the Nairobi slums. According to the recently published Nairobi Cross-Sectional Slum Survey, about 24 percent of women reported unmet need for contraception (APHRC 2014). Jain and colleagues (2013) argue that to effectively reduce unmet need, especially in low- and middle-income countries, programs must not only focus on attracting new users but also need to pay attention to current users and past users who have stopped. Through the "leaking bucket" analogy, Jain (2014) further argues that if the hole in the bucket (past users with unmet need) is not plugged, then the number of women who have abandoned use could exceed the number of current users. Moreover, if method dissatisfaction is not addressed, these concerns might spread to nonusers (Jain 2014; Machiyama and Cleland 2014b).

Choice of contraceptive method after birth in the Nairobi slums was similar to the national method mix. Injectables accounted for 56 percent of all episodes of reversible method use, followed by pills and traditional methods. Implants represented only 4 percent of all episodes, and IUD adoption was even less common. Continued use of injectables is notably higher than use of pills or traditional methods. Nevertheless 40 percent of injectables users had stopped within a year of starting, mainly because of side effects or health concerns. The corresponding figure for pills and traditional methods exceeds 60 percent. This finding echoes that from a national study showing that about 61 percent of married women having unmet need in Kenya had used a modern method in the past, predominantly pills or injectables, and had discontinued the methods because of health concerns or side effects (Machiyama and Cleland 2014a). Because of the poor environment that characterizes the slums, we had expected that access-related factors (including cost, supply, and distance) would be a common reason for discontinuation. However, access-related factors were the least-cited reason (2 percent), which suggests that client-side factors that impede successful use of contraceptive methods also need to be addressed in program planning.

The reproductive consequences of high probability of discontinuation can be offset by prompt switching to an alternative method. Overall, 63 percent of women in our study who stopped using a method because of dissatisfaction or an unspecified reason switched to another method within three months. This estimate is much higher than the national estimate of 35 percent, based on data from the 2003 KDHS (Ali, Cleland, and Shah 2012), a difference that may reflect a high motivation to postpone pregnancy among slum couples. The majority of women who discontinued use of pills, traditional methods, and condoms switched to the dominant method, injectables. Clearly, dissatisfaction with one hormonal method does not discourage women from trying another hormonal method. It is disconcerting that women who stopped using injectables were appreciably less likely to switch than those who discontinued other methods. While 29 percent switched to pills and another 20 percent to other methods, 47 percent of women discontinuing injectables had not switched by month three and 4 percent had become pregnant.

This worrisome trend adds to the growing evidence that the method mix available to women, especially during the postpartum period, may be inadequate to meet their contraceptive needs and hence achieve their fertility goals. The results also suggest inadequate provider counseling on anticipated side effects and choice of method at the point of initiation of a contraceptive method. Expanding the range of methods available and improving client–provider interaction through adequate counseling on potential side effects and management of effects are therefore warranted, especially at the point of initiation, even with switching levels this high.

Our results suggest that encouraging women to adopt long-acting methods may be an effective strategy not only for limiting but also for spacing (Fotso et al. 2013). Our finding that women with four or more living children and those who gave birth at ages 35–49 years were less likely to adopt a method during the 12-month postpartum period underscores the need to provide long-acting or permanent methods (with less method-related dissatisfaction) to regulate fertility, since many of these women may want no more children. The evidence concerning implants is encouraging. Although only 98 episodes of use of this method were observed, discontinuation was markedly lower than for all other methods. Moreover, 4 percent of discontinuers of other methods switched to implants.

Some of the differentials in postpartum behavior are of note. It is surprising that few differences in contraceptive adoption, discontinuation, or switching were observed between women who wanted no more children and those who wanted to postpone the next birth. It appears that both postponement and spacing provide motivation for fertility regulation equal to that of limitation. Less surprising is that better-educated women are more likely than less-educated women to adopt a modern method and more likely to switch after discontinuation. Educational differences in discontinuation were small, suggesting that greater awareness of reproductive biology that is likely associated with length of schooling does not reduce concerns about side effects. Similar results have been reported in other populations (Ali and Cleland 2010).

Nonbreastfeeding women had higher discontinuation rates than women who were breastfeeding at the time of method adoption. Furthermore, women who adopted a method after resumption of menses were also slightly more likely to discontinue than others, perhaps because their motivation was weaker. The concern of many women with the regularity of their menstrual periods and the disruptions that accompany the use of hormonal methods may explain why they stop using these methods (Hindin, McGough, and Adanu 2014; Machiyama and Cleland 2014b).

The need for additional counseling on postpartum fertility was evident in our findings. Survival probabilities show that, by 6 months postpartum, 83 percent of women were sexually active while only 49 percent of women had adopted a method and 49 percent had not resumed menses. These results suggest that many women were partially or fully exposed to the risk of pregnancy but were not using any modern method of contraception. As Ndugwa and colleagues (2011) noted in the baseline analyses for this group of women, special attention needs to be paid to meeting the contraceptive needs of postpartum women, more than half of whom, although sexually active by the sixth month, were not using any contraceptive method but rather awaiting the return of menses to adopt a method. Further, tightly focused research is needed to better understand the link between menses and contraception, which may stem in part from insistence by providers that women prove nonpregnancy by waiting until they are menstruating before seeking a method. It is uncertain whether women understand the small risks of conception during lactational amenorrhea and are prepared to accept them, as Salway and Nurani (1998a, 1998b) found in Bangladesh, or whether they believe that they are fully protected. The postpartum period provides an avenue for intervention as women frequent health centers for child-related care (Rossier and Hellen 2014). The evidence therefore suggests that integrating postpartum family planning with antenatal care and postnatal services is a useful means to reach women and provide accurate information on postpartum fertility, breastfeeding, and options for timing of contraceptive uptake (Townsend 1990; Becker and Ahmed 2001; Borda and Winfrey 2010; Rossier and Hellen 2014). Family planning services that are offered to women at every contact point during antenatal and postnatal periods can be effective in increasing awareness of, demand for, and practice of contraception (Lopez et al. 2012). Integration of postpartum services during antenatal and postnatal visits, if carried out properly, can be an efficient tool to streamline service delivery.

Findings from this study should be interpreted in light of some limitations. Because of the high loss to follow-up, only seven of the ten cohorts had sufficient numbers of women who had 12 months of continuous calendar data. The effect of this attrition is, however, mitigated by the use of 12 months of continuous calendar data. Furthermore, the characteristics of women not retained for analysis due to attrition were not significantly different from the characteristics of women retained for analysis. Second, we assessed only the dynamics of first modern contraceptive use. It is therefore possible that many women who promptly switched to another method have different contraceptive-use dynamics.

Women in Nairobi's slums were generally more likely to discontinue a contraceptive method than other Kenyan women, but they were more likely to switch to an alternative short-acting method. What remains to be investigated is whether switchers experience the same high discontinuation rate as documented for adopters of their first postpartum method. Method-related dissatisfaction explains the high discontinuation rates. Effective interventions that address the health concerns of women with regard to method choice and management of side effects are essential and may increase uptake and continuous use of contraception among women in Nairobi's slums.

## ACKNOWLEDGMENTS

We drew upon data from the Maternal and Child Health Survey, part of the broader study on Urbanization, Poverty, and Health Dynamics funded by the Wellcome Trust (Grant No. GR 07830M). Writing and analysis time for authors was supported by the Strengthening Evidence for Programming on Unintended Pregnancy project (STEP UP, Grant No. SR1109D-6), and general support grants to APHRC from Sida (Grant No. 2011-001578) and the William and Flora Hewlett Foundation (Grant No. 2012–7612).

## References

[sifp38-bib-0001] African Population and Health Research Center (APHRC) . 2002 Population and Health Dynamics in Nairobi's Informal Settlements: Report of the Nairobi Cross‐Sectional Slums Survey (NCSS) 2000, Nairobi.

[sifp38-bib-0002] African Population and Health Research Center (APHRC) . 2014 Population and Health Dynamics in Nairobi's Informal Settlements: Report of the Nairobi Cross‐Sectional Slums Survey (NCSS) 2012, Nairobi.

[sifp38-bib-0003] Ali, Mohamed M. and John Cleland . 2010 “Contraceptive switching after method‐related discontinuation: Levels and differentials,” Studies in Family Planning 41(2): 129–133. 21466113 10.1111/j.1728-4465.2010.00234.x

[sifp38-bib-0004] Ali, Mohamed M. , John Cleland , and Iqbal Shah . 2012 Causes and Consequences of Contraceptive Discontinuation: Evidence from 60 Demographic and Health Surveys. Geneva: World Health Organization.

[sifp38-bib-0005] Ali, Mohamed M. and Iqbal Shah . 2004 “Uptake of contraception following childbirth or pregnancy termination: A preliminary analysis.” Paper presented at the Annual Meeting of the Population Association of America, 1–3 April. Boston, MA.

[sifp38-bib-0006] Becker, Stan and Saifuddin Ahmed . 2001 “Dynamics of contraceptive use and breastfeeding during the post‐partum period in Peru and Indonesia,” Population Studies 55(2): 165–179.

[sifp38-bib-0007] Borda, Maria and William Winfrey . 2010 Postpartum fertility and contraception: An analysis of findings from 17 countries. Baltimore, MD: USAID and Access—Family Planning Initiative.

[sifp38-bib-0008] Fotso, Jean Christophe , Ilene S. Speizer , Carol Mukiira , Paul Kizito , and Vane Lumumba . 2013 Closing the poor‐rich gap in contraceptive use in urban Kenya: Are family planning programs increasingly reaching the urban poor? International Journal for Equity in Health 12: 71. doi: 10.1186/1475‐9276‐12‐71. 23978064 10.1186/1475-9276-12-71 PMC3847584

[sifp38-bib-0009] Gebreselassie, Tesfayi , Shea Oscar Rutstein , and Vinod Mishra 2008 Contraceptive use, breastfeeding, amenorrhea and abstinence during the postpartum period: An analysis of four countries, DHS Analytical Study No. 14. Calverton, MD: Macro International.

[sifp38-bib-0010] Hindin, Michelle J. , Laura J. McGough , and Richard M. Adanu . 2014 “Misperceptions, misinformation and myths about modern contraceptive use in Ghana,” Journal of Family Planning and Reproductive Health Care 40(1): 30–35. 23771916 10.1136/jfprhc-2012-100464

[sifp38-bib-0011] Jain, Anrudh . 2014 The leaking bucket phenomenon in family planning. Guest post. http://champions4choice.org/2014/09/the-leaking-bucket-phenomenon-in-family-planning/.

[sifp38-bib-0012] Jain, Anrudh K. , Francis Obare , Saumya RamaRao , and Ian Askew . 2013 “Reducing unmet need by supporting women with met need,” International Perspectives on Sexual and Reproductive Health 39(3): 133–41. 24135045 10.1363/3913313

[sifp38-bib-0013] Kennedy, Kathy I. and Cynthia M. Visness . 1992 “Contraceptive efficacy of lactational amenorrhoea,” The Lancet, 339(8787): 227–230. 10.1016/0140-6736(92)90018-x 1346183

[sifp38-bib-0014] Kenya National Bureau of Statistics (KNBS) and ICF Macro 2010 Kenya Demographic and Health Survey 2008–09. Calverton, MD: KNBS and ICF Macro.

[sifp38-bib-0015] Lopez, Laureen M. , Janet E. Hiller , David A. Grimes , and Mario Chen . 2012 Education for contraceptive use by women after childbirth, Cochrane Database of Systematic Reviews. 10.1002/14651858.CD001863.pub3 22895923

[sifp38-bib-0016] Machiyama, Kazuyo and John Cleland . 2014a Insights into unmet need in Kenya, “*STEP UP Research Report*. London School of Hygiene & Tropical Medicine, London.

[sifp38-bib-0017] Machiyama, Kazuyo . 2014b “Unmet need for family planning in Ghana: The shifting contributions of lack of access and attitudinal resistance,” Studies in Family Planning 45(2): 203–226. 24931076 10.1111/j.1728-4465.2014.00385.x

[sifp38-bib-0018] MCHIP and USAID . 2012 Family planning needs during the first two years postpartum in Kenya. http://www.mchip.net/sites/default/files/Kenya%20DHS%20Reanalysis%20for%20PPFP_final.pdf.

[sifp38-bib-0019] Ndugwa, Robert P. , John Cleland , Nyovani J. Madise , Jean‐Christophe Fotso , and Eliya M. Zulu . 2011 “Menstrual pattern, sexual behaviors, and contraceptive use among postpartum women in Nairobi urban slums,” Journal of Urban Health, 88(Suppl. 2): 341–355. 10.1007/s11524-010-9452-6 PMC3132236 20449772

[sifp38-bib-0020] Ross, John A. and William L. Winfrey . 2001 “Contraceptive use, intention to use and unmet need during the extended postpartum period,” International Family Planning Perspectives 27(1): 20–27.

[sifp38-bib-0021] Rossier, Clémentine and Jacqueline Hellen . 2014 “Traditional birthspacing practices and uptake of family planning during the postpartum period in Ouagadougou: Qualitative results,” International Perspectives on Sexual and Reproductive Health 40(2): 87–94. 25051580 10.1363/4008714

[sifp38-bib-0022] Rutstein, Shea Oscar and Rebecca Winter 2014 The effects of fertility behavior on child survival and child nutritional status: Evidence from the Demographic and Health Surveys 2006 to 2012, DHS Analytical Study No. 37. Rockville, MD: ICF International.

[sifp38-bib-0023] Salway, Sarah and Sufia Nurani . 1998a “Postpartum contraceptive use in Bangladesh: Understanding users' perspectives,” Studies in Family Planning 29(1): 41–57. 9561668

[sifp38-bib-0024] V. 1998b “Uptake of contraception during postpartum amenorrhoea: Understandings and preferences of poor, urban women in Bangladesh,” Social Science & Medicine 47(7): 899–909. 9722110 10.1016/s0277-9536(98)00154-3

[sifp38-bib-0025] Sedgh, Gilda and Rubina Hussain . 2014 “Reasons for contraceptive nonuse among women having unmet need for contraception in developing countries,” Studies in Family Planning 45(2): 151–169. 24931073 10.1111/j.1728-4465.2014.00382.x

[sifp38-bib-0026] Singh, Susheela , Jacqueline E. Darroch , Lori S. Ashford , and Michael Vlassoff 2009 Adding it up: The costs and benefits of investing in family planning and maternal and newborn health. New York: Guttmacher Institute and United Nations Population Fund.

[sifp38-bib-0027] Townsend, S. 1990 Postpartum contraception: Developing strategies for expanded services. Research Triangle Park, NC: Network 11 (3): **1**, 8–9, 15. 12342902

[sifp38-bib-0028] Tsui, Amy and Andreea A. Creanga 2009 “Does contraceptive use reduce neonatal and infant mortality? Findings from a multi‐country analysis.” Paper presented at the Annual Meeting of the Population Association of America, Detroit, Michigan.

[sifp38-bib-0029] Warren, Charlotte E. , Timothy Abuya , and Ian Askew . 2013 Family planning practices and pregnancy intentions among HIV‐positive and HIV‐negative postpartum women in Swaziland: A cross sectional survey, BMC Pregnancy and Childbirth 13: 150. 23855776 10.1186/1471-2393-13-150 PMC3720191

[sifp38-bib-0030] Westoff, Charles F. 2001 Unmet Need at the End of the Century. DHS Comparative Reports No. 1. Calverton, MD: Macro International.

[sifp38-bib-0031] Winfrey, William and Kshitiz Rakesh 2014 Use of family planning in the postpartum period, DHS Comparative Reports 36. Rockville, MD: ICF International.

[sifp38-bib-0032] Ziraba, Abdhalah , Nyovani Madise , Samuel Mills , Catherine Kyobutungi , and Alex Ezeh . 2009 “Maternal mortality in the informal settlements of Nairobi City: What do we know?” Reproductive Health 6 6: 4755–4756. 10.1186/1742-4755-6-6 PMC2675520 19386134

